# The Triglyceride/HDL Ratio as a Non-Invasive Marker for Early-Stage NAFLD: A Retrospective Cross-Sectional Study of 2588 Patients

**DOI:** 10.3390/diagnostics15162045

**Published:** 2025-08-14

**Authors:** Emre Hoca, Bilal Cangir, Süleyman Ahbab, Seher İrem Şahin, Ece Çiftçi Öztürk, Ayşe Öznur Urvasızoğlu, Nilsu Kalaycı, İsmail Engin, Hayriye Esra Ataoğlu

**Affiliations:** 1 Department of Internal Medicine, Haseki Training and Research Hospital, University of Health Sciences, Istanbul 34260, Turkey; drsahbab@gmail.com (S.A.); siremcetin@gmail.com (S.İ.Ş.); eciftci3506@gmail.com (E.Ç.Ö.); dr.nilsukalayci@gmail.com (N.K.); eataoglu@gmail.com (H.E.A.); 2 Department of Internal Medicine, Başakşehir Çam ve Sakura City Hospital, Istanbul 34480, Turkey; drbilalcangir@gmail.com; 3 Department of Endocrinology, İstanbul Şişli Hamidiye Etfal Training and Research Hospital, University of Health Sciences, Istanbul 34093, Turkey; oznurvasiz@gmail.com; 4 Department of Endocrinology, Training and Research Hospital, Ümraniye, University of Health Sciences, Istanbul 34764, Turkey; ismailengin1987@gmail.com

**Keywords:** dyslipidemia, HDL cholesterol, non-alcoholic fatty liver disease, triglyceride, triglyceride/HDL ratio

## Abstract

**Background**: Non-alcoholic fatty liver disease (NAFLD) is a global public health issue. Although liver biopsy remains the gold standard for diagnosing hepatosteatosis, its invasiveness, high cost, and associated risks limit its widespread use. Therefore, there is a need for reliable, non-invasive, and cost-effective biomarkers to aid in the early detection of NAFLD. Our objective was to determine the utility of the triglyceride (TG)-to-high-density-lipoprotein (HDL) ratio in predicting non-alcoholic fatty liver disease. **Methods**: This retrospective cross-sectional study included 2588 patients who met the inclusion criteria. Demographic data and laboratory results were collected from electronic health records. Experienced radiologists performed abdominal ultrasonography to assess fatty liver according to the EASL 2021 criteria. The TG/HDL ratio and other non-invasive scores (APRI, FIB-4, ALT/AST, TG/glucose) were calculated. Early-stage disease was defined as grade 1 or grade 2 hepatosteatosis. **Results**: The TG/HDL ratio was significantly higher in NAFLD patients (AUROC: 0.682) and outperformed the other non-invasive indices. At the optimal cut-off value of 1.86, the sensitivity was 80.7%, and the specificity was 45.5%. The TG/HDL ratio correlated positively with markers of glycemic control, inflammation, and liver enzymes. **Conclusions**: The TG/HDL ratio is an accessible and valuable parameter for predicting non-alcoholic fatty liver disease. It offers a non-invasive alternative to liver biopsy and potentially prevents complications from non-alcoholic fatty liver disease or diagnostic approaches.

## 1. Introduction

Non-alcoholic fatty liver disease (NAFLD) is becoming increasingly prevalent globally [1,2]. This is due to the increasing prevalence of metabolic syndrome and obesity, as well as changes in dietary habits. The true prevalence of NAFLD is unknown, as it is the most common (approximately 90%) cause of asymptomatic aspartate aminotransferase (AST) and alanine aminotransferase (ALT) elevation in patients presenting to clinics, and the majority of cases are asymptomatic [1]. In some screening-based studies conducted in the adult population, the prevalence of NAFLD was found to be 17–33% (75% in obese patients), and the prevalence of non-alcoholic steatohepatitis (NASH) was approximately 3% [3]. NAFLD is not only confined to the liver but is increasingly recognized as a hepatic manifestation of systemic metabolic dysfunction and is closely associated with a significantly increased risk of type 2 diabetes mellitus, insulin resistance, obesity, and cardiovascular diseases [4]. NAFLD is characterized by fat accumulation in the liver and is the most common cause of chronic liver disease. A sedentary life, certain drugs (such as steroid hormones, tamoxifen, and cisplatin), and environmental and genetic factors may also play a role in the development of NAFLD [5].

For a diagnosis of NAFLD, steatosis should be demonstrated on radiological imaging or tissue histology; causes that may lead to hepatic steatosis should be excluded; and excessive alcohol consumption, as well as other causes of chronic liver disease, should be ruled out [6]. The gold standard in the diagnosis of hepatosteatosis is liver biopsy. However, it is mostly not preferred for the diagnosis of hepatosteatosis because it is an invasive procedure, is not cost-effective, and involves complications such as bleeding or liver injury. Therefore, non-invasive or less invasive techniques are used to diagnose hepatosteatosis whenever possible [7]. Imaging techniques commonly used to diagnose NAFLD include abdominal ultrasound (US) imaging, computed tomography (CT), and magnetic resonance imaging (MRI). US is often the preferred imaging method because it is easily accessible and does not contain ionizing radiation [8].

Although the term non-alcoholic fatty liver disease has recently been redefined as metabolic-dysfunction-associated steatotic liver disease (MASLD), this study maintains the NAFLD terminology, as it remains commonly used in clinical practice and the literature, particularly in settings where complete metabolic profiling is not feasible.

Fibrosis may develop against a background of NASH in the later stages of the disease, and the risk of liver cirrhosis and hepatocellular carcinoma may increase in advanced cases. Therefore, detecting fibrosis in NAFLD patients in the early stages and determining the appropriate treatment strategies are essential. In addition to invasive methods such as liver biopsy, non-invasive scores such as the AST-to-platelet ratio index (APRI), fibrosis 4 (FIB-4) score, and ALT-to-AST ratio are also used to evaluate fibrosis in NAFLD. These scores are simple, non-invasive tests widely used in routine clinical practice and aid clinical decision-making to assess liver fibrosis in the foreground. Studies show that these scores can predict hepatic fibrosis and NAFLD [9,10].

Studies have shown that the ratio of different lipid parameters such as the LDL cholesterol/HDL cholesterol ratio and remnant cholesterol/HDL cholesterol ratio can be used to predict NAFLD or its stage [11,12]. Hypertriglyceridemia and low high-density lipoprotein (HDL) cholesterol levels play an essential role in the pathophysiology of NAFLD and are components of metabolic syndrome. Intense triglyceride (TG) accumulation is observed in the hepatocytes, especially in patients with NAFLD. Our study aimed to determine whether the TG-to-HDL ratio is superior to other scoring systems in predicting NAFLD and whether this ratio can be used rapidly and reliably to diagnose and follow hepatosteatosis.

## 2. Patients and Methods

### 2.1. The Study Participants

**Figure 1:**
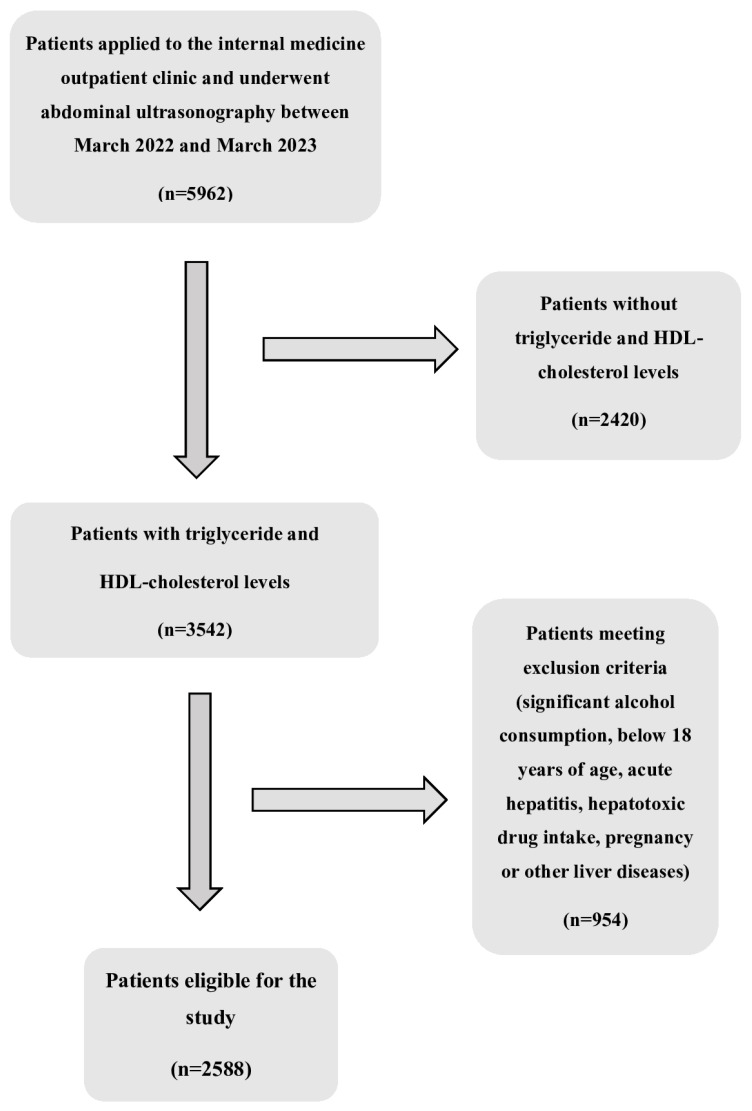
Flow diagram of participants.

This study had a retrospective, cross-sectional study design. The University of Health Sciences, Haseki Training and Research Hospital’s institutional ethics committee reviewed and approved the study protocol (reference number: 02-2023; date of approval: 11 January 2023). Also, the ethics committee anonymized and approved the database information with no need for consent. Data from electronic health records and laboratory databases for 5962 patients admitted to Haseki Training and Research Hospital’s Internal Medicine outpatient clinic between March 2022 and March 2023 who underwent abdominal ultrasonography were analyzed retrospectively. The exclusion criteria included significant alcohol intake (≥140 g/week ethanol consumption for women, ≥210 g/week ethanol consumption for men), being under 18 years of age, hepatotoxic drug or anti-hyperlipidemic agent usage, acute hepatitis, pregnancy, and having a disease affecting the liver. Cases with incomplete laboratory or ultrasonographic data were excluded from the analysis. The inclusion criteria were being over 18 years of age, available fasting triglyceride and HDL measurements, and ultrasonographic evidence of being with or without hepatic steatosis. A total of 2588 patients who fulfilled the necessary criteria were included in this study, as seen in the flow diagram for the study (Figure 1). Due to the retrospective design of this study and the unavailability of certain clinical data, such as body mass index (BMI), blood pressure, and antihypertensive medication use, we were unable to apply the updated MASLD criteria. Therefore, the diagnostic classification was based on ultrasonographic evidence of hepatic steatosis in accordance with the traditional definition of NAFLD.

### 2.2. The Laboratory and Radiological Analysis

Demographic data (such as age and gender) and clinical characteristics (comorbidities and medications used) were recorded. The test values (AST, ALT, TG, HDL cholesterol, total cholesterol, complete blood count, HbA1c, albumin, glucose, creatinine, uric acid, and hemograms) obtained after fasting for at least eight hours were recorded. The TG/HDL ratio was calculated by dividing serum triglyceride (mg/dL) by high-density lipoprotein cholesterol (mg/dL). Similarly, the APRI, FIB-4, and ALT/AST and TG/glucose ratios were calculated using the standard formulas. A ROC analysis was used to identify the diagnostic performance of each index. The optimal cut-off value was determined using the Youden Index. Hepatic steatosis was evaluated semiquantitatively through abdominal ultrasonography performed by experienced radiologists blinded to the clinical and laboratory data. Patients with grade 1 or higher hepatic steatosis were classified as NAFLD-positive. An NAFLD diagnosis was based on the presence of hepatic steatosis according to the European Association for the Study of the Liver (EASL) 2021 criteria, after the exclusion of secondary causes and significant alcohol intake. To characterize early-stage NAFLD, we evaluated the distribution of hepatic steatosis grades among the NAFLD patients. Based on ultrasonography reports, the majority of the patients (%97) were classified as having grade 1 (*n* = 878) or grade 2 (*n* = 411) steatosis, indicating that our cohort predominantly represented early-stage disease.

### 2.3. The Statistical Analysis

The statistical analysis was performed using SPSS 26.0 for Windows (IBM Corp.: Armonk, NY, USA). Numeric values were expressed as the mean ± standard deviation. Normality was assessed using descriptive statistics and the Kolmogorov–Smirnov test in combination with skewness and kurtosis parameters to allow for both a statistical and visual evaluation, as is particularly suitable for large datasets. Normally distributed variables were assessed using a *t*-test, and irregular variables were evaluated using the Mann–Whitney U test. Comparisons of numerical variables in more than two groups were made using the one-way ANOVA test if there was a normal distribution or the Kruskal–Wallis test if there was no normal distribution. Subgroup analyses were interpreted using Bonferroni correction. Categorical variables were evaluated with the chi-square test. The sensitivity and specificity values for non-invasive scoring and the triglyceride/HDL ratio for predicting hepatosteatosis were calculated. Receiver operating characteristic (ROC) analyses were performed to determine the diagnostic accuracy of the related laboratory results and ratios. The optimal cut-off value for the Tg/HDL ratio was determined using the Youden Index. To evaluate whether a combination of non-invasive markers could improve the diagnostic accuracy for NAFLD, a binary logistic regression model was constructed. Independent variables included FIB-4, the APRI, the Tg/HDL ratio, the triglyceride/glucose ratio, and the ALT/AST ratio. The predicted probabilities from this model were subsequently used to generate a combined ROC curve. Odds ratios (ORs), confidence intervals, and statistical significance levels were calculated for each variable, and the model’s overall performance was assessed via the area under the curve (AUC). Pearson’s correlation coefficient was used to assess the linear relationships between variables, while Spearman’s correlation was used to evaluate the monotonic relationships. Descriptive statistics were presented as the mean ± standard deviation, and *p*-values ≤ 0.05 were considered significant.

## 3. Results

Of the patients included in the study, 1631 were female, and 957 were male. A total of 811 female and 459 male patients did not have NAFLD, while 820 female and 498 male patients had NAFLD. There was no statistically significant difference in the incidence of NAFLD between genders (*p*: 0.249). The mean age of the patients was 45.31 ± 12.50 years, 49.07 ± 10.34 years in the group with NAFLD and 41.42 ± 13.33 years in the group without hepatosteatosis. When the grades of hepatosteatosis were evaluated, it was seen that 878 patients had grade 1 hepatosteatosis, 411 patients had grade 2 hepatosteatosis, and 29 patients had grade 3 hepatosteatosis, out of 1318 patients with NAFLD.

Among the laboratory parameters, the white blood cell (WBC), neutrophil count, glucose, HbA1c, AST, ALT, alkaline phosphatase (ALP), lactate dehydrogenase (LDH), albumin, total cholesterol, TG, and C-reactive protein (CRP) values were significantly higher in the patients with NAFLD, and the HDL values were significantly higher in the patients without hepatosteatosis (Table 1). There was no statistically significant correlation between platelet (PLT) count and the presence or absence of hepatosteatosis.

**Figure 2:**
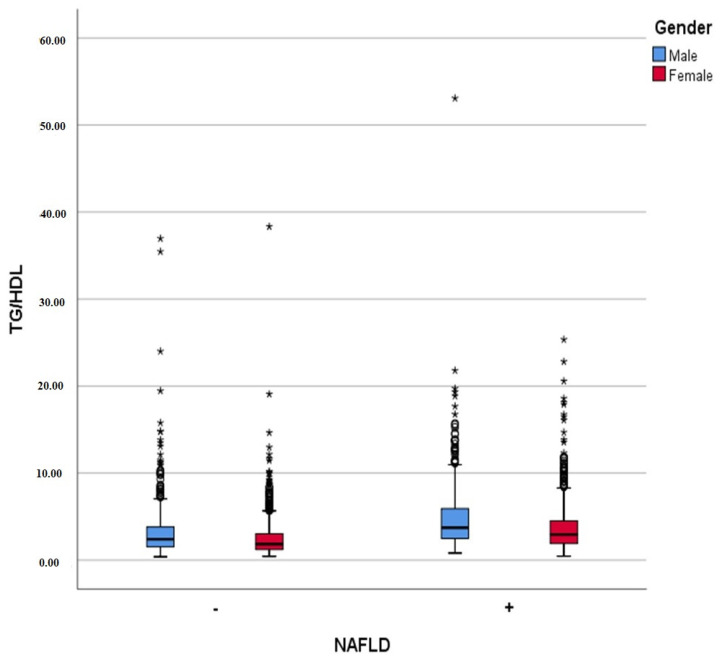
Boxplot graphic of TG/HDL ratio according to NAFLD presence and gender. Asterisks (*) indicate extreme outliers, defined as values greater than 1.5 times the interquartile range above the upper quartile.

When the patients were analyzed according to the non-invasive disease assessment methods, the triglyceride/HDL ratios, APRI scores, FIB-4 scores, ALT/AST ratios, and TG/glucose ratios were significantly higher in the patients with NAFLD (all *p*-values: <0.001) (Table 2). In the subgroup analysis according to gender, the TG/HDL ratio was 2.48 ± 2.32 (median: 1.84) in the patients without NAFLD and 3.64 ± 2.75 (median: 2.94) in the patients with NAFLD. In men, this ratio was 3.31 ± 3.49 (median: 2.39) in those without NAFLD and 4.81 ± 3.96 (median: 3.72) in those with NAFLD (Figure 2). All of these differences were statistically significant (*p* < 0.001).

**Figure 3:**
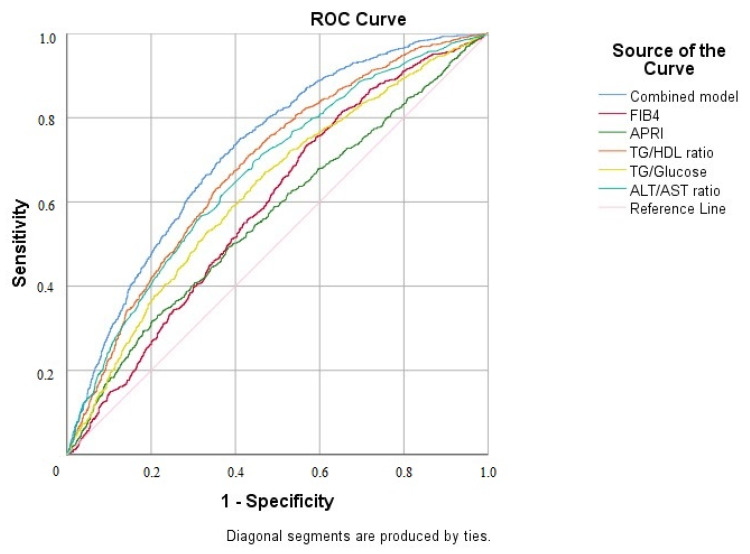
ROC curve analysis of non-invasive parameters for predicting NAFLD.

In the multivariate analysis, the TG/HDL ratio (OR: 1.12, 95% CI: 1.05–1.19), FIB-4 (OR: 2.08, 95% CI: 1.64–2.64), the APRI (OR: ~0, 95% CI: not estimable due to model limitations), and the ALT/AST ratio (OR: 4.27, 95% CI: 3.36–5.41) remained independently associated with NAFLD (Table 3). When the ROC analysis was performed to evaluate the predictive value of the TG/HDL ratio and the other non-invasive parameters for NAFLD, the AUROC for the TG/HDL ratio was 0.682 (95% CI: 0.662–0.703), and this ratio outperformed the APRI (0.565), FIB-4 (0.591), and the ALT/AST ratio (0.668). A combined logistic regression model including TG/HDL, FIB-4, APRI, ALT/AST, and TG/glucose achieved a higher diagnostic performance, with an AUROC of 0.723 (95% CI: 0.704–0.743) (Figure 3 and Table 4). The optimal cut-off value for the TG/HDL ratio was identified as 1.86 using the Youden Index, yielding a sensitivity of 80.7% and a specificity of 45.5% for NAFLD detection (Table 5).

As shown in Table 6, when the correlation of the TG/HDL ratio with the other biochemical findings and parameters was analyzed, it was observed that it was positively correlated with all of the parameters except for FIB-4, LDH, and PLT count.

## 4. Discussion

The findings of this study demonstrate the clinical relevance of the TG/HDL ratio as a practical tool in the early identification of NAFLD. The observed AUROC of 0.682, with high sensitivity at a low cut-off value, supports its utility, particularly in identifying early-stage disease, such as grade 1 steatosis, which comprised the majority of our NAFLD cohort.

Non-alcoholic fatty liver disease (NAFLD) is an increasingly common health problem not only in Turkey but also worldwide due to the increasing prevalence of metabolic syndrome, obesity, and changes in dietary habits. Current studies suggest that the global prevalence of NAFLD is approximately 25% [13]. Imaging methods are most commonly used to diagnose NAFLD. However, routine imaging may delay diagnosis and contribute to unnecessary healthcare costs, and patients are exposed to unnecessary radiation in methods such as computed tomography. In patients with suspected NAFLD, it is essential that a simple value such as the TG/HDL ratio, which is readily derived from routine laboratory tests, may assist in the diagnosis. In this way, both losses of time and labor can be prevented.

The review by Younossi ZM and the study by Tabacu L et al. reported that the incidence of NAFLD increased with age [14,15]. Similarly, our study found that the mean age of the patients with hepatosteatosis was higher than that of those without hepatosteatosis.

In a study conducted by Summart et al. in Thailand, it was observed that the prevalence of NAFLD was higher in women (especially in the postmenopausal period) than in men [16]. In a study conducted by Eguchi et al. in Japan and published in 2012, it was observed that the incidence of NAFLD in men was approximately three times higher than that in women [17]. In our study, although the prevalence was higher in men, no statistically significant difference was observed in NAFLD prevalence between genders. Gender-related differences in NAFLD prevalence likely result from both biological and sociocultural factors. Estrogen may confer a protective effect in premenopausal women by enhancing insulin sensitivity and reducing hepatic fat accumulation. In contrast, men tend to have more visceral adiposity, which is strongly linked to hepatic steatosis. Lifestyle differences, such as dietary habits and healthcare utilization patterns, may also contribute to the observed disparity between genders.

Increased hepatic insulin resistance plays a vital role in the pathophysiology of NAFLD. Numerous studies and reviews have demonstrated that metabolic syndrome and diabetes mellitus are more common pathologies in patients with NAFLD compared to the average population [14,18]. In a meta-analysis by Ballestri et al. in which the data for 117,020 patients from 20 different studies were evaluated, it was concluded that the incidence of type 2 DM was approximately doubled in patients with NAFLD [19]. Similarly, our study found that the glucose and HbA1c values were significantly higher in patients with hepatosteatosis and were positively correlated with the TG/HDL ratio.

It has been reported that serum aminotransferase (AST and ALT) levels may be slightly elevated in patients with hepatosteatosis secondary to hepatocyte inflammation [20,21]. The presence of these values in the normal range does not exclude the disease. In our evaluation, AST and ALT were significantly increased in the patients with hepatosteatosis. In addition, studies are showing that the ALT/AST ratio is a parameter that can be used to support the diagnosis of NAFLD [9,22,23]. Some studies have shown that aminotransferase levels, primarily ALT, increase as fibrosis increases in NAFLD patients [24]. Because of this increase in ALT, which increases more precisely and more markedly than AST under hepatocyte damage, the ALT/AST ratio has been found to be higher in patients with hepatosteatosis than that in those without hepatosteatosis. Our analysis found that the ALT/AST ratio was higher in patients with NAFLD in a manner that supported the data from the literature. Similarly, the inflammation indicators of the WBC count and CRP were higher in the NAFLD patients due to inflammation in the hepatocytes and were positively correlated with the TG/HDL ratio. These findings also supported previous studies [25].

Dyslipidemia, one of the components of metabolic syndrome, frequently accompanies NAFLD. Studies have shown that significantly elevated triglycerides and low HDL levels are observed more frequently in patients with hepatosteatosis [26,27]. Increased triglyceride accumulation in the hepatocytes is considered one of the main pathophysiological mechanisms in NAFLD [26]. In support of these findings, Li et al. found that the triglyceride levels were higher and HDL cholesterol levels were lower in patients with hepatosteatosis than these levels in those without NAFLD [27]. In addition, some studies show that the triglyceride/HDL ratio is higher in patients with hepatosteatosis than that in the average population [28,29,30,31,32]. A study by Wu et al. showed that an increase in the triglyceride/HDL ratio was associated with an increase in the stage of hepatosteatosis, especially with advanced hepatosteatosis [30]. Similar results are available between total cholesterol levels and NAFLD. In two different studies conducted with patients with chronic hepatitis B and type 2 diabetes mellitus, the total cholesterol levels and total cholesterol/HDL cholesterol ratios were found to be higher in patients with hepatosteatosis, similar to our study [33,34].

The search for parameters that can rapidly and accurately detect NAFLD, a global health problem, continues. Although histological confirmation remains the diagnostic gold standard for NAFLD, its invasive nature, associated risks, and ethical considerations limit its use in large-scale or retrospective studies. In contrast, abdominal ultrasonography offers a practical and non-invasive alternative. A comprehensive meta-analysis by Hernaez et al., which included 49 studies, demonstrated that ultrasonography has a pooled sensitivity of 84.8% and specificity of 93.6% for detecting moderate to severe hepatic steatosis when compared with histology [8]. These findings support its reliability in epidemiological and clinical research settings. Accordingly, our study utilized ultrasonographic diagnosis in alignment with the European Association for the Study of the Liver (EASL) 2021 guidelines, and evaluations were performed by experienced radiologists blinded to the clinical and laboratory data to reduce observer bias. Therefore, studies have been conducted to evaluate whether non-invasive parameters such as the APRI, FIB-4, and the ALT/AST ratio, which were mainly developed to predict fibrosis in the liver in daily practice, are also predictive of NAFLD, and many different results have been found. In a study conducted by Sayar et al. in patients with chronic hepatitis B infections, it was observed that a high APRI score was not associated with the presence of NAFLD [35]. In our study, the TG/HDL ratios were significantly higher in the patients with NAFLD. A study conducted by Yin et al. evaluating high-risk metabolic-dysfunction-related steatohepatitis patients showed that the APRI had a sensitivity of 24.7% and FIB-4 had a sensitivity of 26.9% for steatohepatitis [36]. Another study conducted in Portugal observed that the APRI had a sensitivity of 65.4% and FIB-4 had a sensitivity of 61.5% at different cut-off values [37].

Similarly, the ALT/AST ratio was thought to be usable for predicting NAFLD like the other non-invasive parameters. However, it was observed that its sensitivity remained as low as 55% at the determined cut-off value [38]. In our study, when the TG/HDL ratio was evaluated according to the cut-off value, it was found that the TG/HDL ratio’s sensitivity for NAFLD was higher compared to other studies’ findings. Considering that the majority of the patient group in our study had grade 1 hepatosteatosis, the TG/HDL ratio seems to be an accessible and helpful parameter in predicting NAFLD, especially in the initial stage.

Our findings demonstrate that the TG/HDL ratio is a valuable predictor of NAFLD. This non-invasive parameter offers a practical alternative to liver biopsy, reducing the risks and costs associated with invasive procedures. The TG/HDL ratio can be easily calculated from routine laboratory tests, making it an accessible tool for the early diagnosis and management of NAFLD. From a public health perspective, the TG/HDL ratio could be integrated into primary care screening algorithms, particularly for high-risk populations such as individuals with obesity, diabetes, or metabolic syndrome. Its simplicity may allow for earlier identification of hepatic steatosis, enabling preventive lifestyle interventions and targeted follow-up. Although the AUROC of 0.682 reflects moderate accuracy, it is comparable with traditional indices such as the APRI and FIB-4, reinforcing the practical utility of TG/HDL as a simple screening tool. Our findings also suggest that while the TG/HDL ratio is an independently valuable marker for predicting NAFLD, its diagnostic performance could increase when used in combination with other non-invasive indices such as FIB-4, the APRI, and ALT/AST. This supports the idea that multi-marker approaches may offer greater sensitivity and specificity in the non-invasive assessment of hepatic steatosis. While promising, the current findings require validation in prospective cohorts and in populations with histologic confirmation of NAFLD. Further research may also clarify sex-specific thresholds or utility in pediatric and geriatric groups.

The large sample size and the use of multiple validated indices strengthen the reliability of our findings. Also, several limitations should be acknowledged. The reliance on ultrasonographic findings without histological confirmation may have introduced variability into the diagnosis of NAFLD, especially in mild steatosis. Also, ultrasonography cannot be used to assess fibrosis. Due to the retrospective nature of this study, data regarding the patients’ dietary habits, physical activity levels, and specific medication use (e.g., statins, antidiabetics) were not consistently available in the medical records and thus could not be included in the analysis. These factors are indeed relevant to both lipid metabolism and NAFLD progression and should be considered in future prospective studies. Although recent international consensus statements have introduced MASLD terminology to emphasize the metabolic basis of fatty liver disease, the use of NAFLD remains practical in retrospective studies and routine clinical settings where complete metabolic data are lacking. Additionally, although we could not directly apply the MASLD criteria due to missing variables, our results showed that some metabolic indicators (e.g., the TG/glucose ratio and fasting glucose) were significantly higher in the patients with NAFLD. This indirectly supports the metabolic component emphasized in MASLD and strengthens the clinical relevance of our findings. Thus, our findings add to the evidence for NAFLD while recognizing the appropriateness of the term MASLD for prospective research. In addition, as a retrospective cross-sectional study, our findings are limited to associations rather than causation. However, the large sample size and the use of multiple non-invasive indices provide a valuable snapshot of TG/HDL’s diagnostic potential in routine practice.

## 5. Conclusions

Our study confirms the utility of the TG/HDL ratio as a non-invasive biomarker for hepatic steatosis. Given its simplicity and strong association with NAFLD in our cohort, the TG/HDL ratio holds promise as an accessible adjunctive tool in primary screening protocols, particularly when imaging or biopsy is not feasible. When combined with other non-invasive parameters such as FIB-4 and ALT/AST, its diagnostic performance improves considerably. Although more studies are needed on this subject, its widespread use could enhance early diagnosis and management, potentially reducing NAFLD-related complications, especially in patients at risk of hepatosteatosis. Future studies incorporating histologic endpoints and longitudinal outcomes will be critical to establishing the prognostic value of this biomarker.

## Figures and Tables

**Table 1:** Descriptive characteristics and laboratory findings for all patients, NAFLD and non-NAFLD groups.

Characteristics and Findings	All Patients (*n*: 2588, Mean ± Std)	Patients Without NAFLD (*n*: 1270, Mean ± Std)	Patients with NAFLD (*n*: 1318, Mean ± Std)	*p*-Value
Age (years)	45.31 ± 12.50	41.42 ± 13.33	49.07 ± 10.34	**<0.001**
Gender (F/M)	1631/957	811/459	820/498	0.249
WBCs (10^9^/L)	7.35 ± 1.93	7.11 ± 1.91	7.58 ± 1.92	**<0.001**
Neutrophil (10^9^/L)	4.32 ± 1.50	4.23 ± 1.56	4.40 ± 1.45	**0.005**
Hemoglobin (g/L)	13.58 ± 1.78	13.4 ± 1.85	13.76 ± 1.70	**<0.001**
Platelets (10^9^/L)	258.81 ± 70.92	257.26 ± 76.02	260.31 ± 65.62	0.275
Glucose (mg/dL)	109.34 ± 41.40	100.63 ± 31.48	117.73 ± 47.61	**<0.001**
HbA1c (%)	6.25 ± 1.48	5.88 ± 1.30	6.52 ± 1.54	**<0.001**
AST (U/L)	24.80 ± 38.88	22.67 ± 30.62	26.84 ± 45.33	**<0.001**
ALT (U/L)	29.91 ± 49.99	24.02 ± 36.87	35.60 ± 59.45	**<0.001**
ALP (IU/L)	84.37 ± 36.09	82.38 ± 42.0	86.45 ± 28.49	**0.013**
LDH (U/L)	182.75 ± 47.96	178.05 ± 46.45	187.77 ± 49.06	**<0.001**
Creatinine (mg/dL)	0.75 ± 0.16	0.74 ± 0.15	0.76 ± 0.16	**<0.001**
Triglyceride (mg/dL)	147.42 ± 93.89	122.67 ± 80.57	171.27 ± 99.50	**<0.001**
HDL-cholesterol (mg/dL)	49.28 ± 12.82	51.26 ± 13.54	47.37 ± 11.78	**<0.001**
Total cholesterol (mg/dL)	189.23 ± 41.0	181.16 ± 40.62	197.02 ± 39.88	**<0.001**
Uric acid (mg/dL)	4.70 ± 1.31	4.34 ± 1.22	5.06 ± 1.30	**<0.001**
Albumin (g/L)	45.63 ± 3.0	45.41 ± 3.28	45.83 ± 2.70	**0.002**
CRP (mg/L)	4.91 ± 9.32	4.31 ± 9.85	5.49 ± 8.75	**0.007**

*n*: number of patients; std: standard deviation; NAFLD: non-alcoholic fatty liver disease; F: female; M: male; WBC: white blood cell; HbA1c: hemoglobin A1c; AST: aspartate aminotransferase; ALT: alanine aminotransferase; ALP: alkaline phosphatase; LDH: lactate dehydrogenase; HDL: high-density lipoprotein; CRP: c-reactive protein. *p* < 0.05: statistically significant; significant values are shown in bold.

**Table 2:** Association of non-invasive parameters with NAFLD.

	Patients Without NAFLD (*n*: 1270)	Patients with NAFLD (*n*: 1318)	*p*-Value
**TG/HDL ratio**	2.79 ± 2.83	4.08 ± 3.30	**<0.001**
**ALT/AST ratio**	0.99 ± 0.39	1.23 ± 0.49	**<0.001**
**APRI**	0.32 ± 0.64	0.35 ± 0.62	**<0.001**
**FIB-4 score**	0.85 ± 0.80	0.93 ± 0.50	**<0.001**
**TG/Glucose ratio**	1.24 ± 0.74	1.54 ± 0.88	**<0.001**

*n*: number of patients; TG: triglyceride; HDL: high-density lipoprotein; ALT: alanine aminotransferase; APRI: AST-to-platelet ratio index; FIB-4: fibrosis 4. *p* < 0.05: statistically significant; significant values are shown in bold.

**Table 3:** Multivariate logistic regression analysis for predictors of NAFLD.

	B	S.E.	Odds Ratio	*p*
(Constant)	−2.518	0.188	0.081	
FIB-4	0.734	0.116	2.082	**<0.001**
APRI	−61.141	14.798	0	**<0.001**
Tg/HDL ratio	0.112	0.03	1.118	**<0.001**
Tg/glucose ratio	0.101	0.095	1.107	0.288
ALT/AST ratio	1.452	0.122	4.27	**<0.001**

*p* < 0.05: statistically significant values are shown in bold.

**Table 4:** AUROC and %95 CI of non-invasive parameters for predicting NAFLD.

	AUROC for NAFLD (%95 CI)	*p*-Value
**TG/HDL ratio**	0.682 (0.662–0.703)	**<0.001**
**Combined model**	0.723 (0.704–0.743)	**<0.001**
**ALT/AST ratio**	0.668 (0.647–0.689)	**<0.001**
**APRI**	0.565 (0.543–0.587)	**<0.001**
**FIB-4**	0.591 (0.569–0.613)	**<0.001**
**TG/glucose ratio**	0.626 (0.604–0.647)	**<0.001**

TG: triglyceride; HDL: high-density lipoprotein; ALT: alanine aminotransferase; APRI: AST-to-platelet ratio index; FIB-4: fibrosis 4. *p* < 0.05: statistically significant; significant values are shown in bold.

**Table 5:** The distribution of the patients according to the determined cut-off value for the TG/HDL ratio.

TG/HDL Ratio	*N* of Patients Without NAFLD	*N* of Patients with NAFLD	Total	*p*-Value
**<1.86**	578	254	832	**<0.001**
**≥1.86**	692	1064	1756	
**Total**	1270	1318	2588	

TG: triglyceride; HDL: high-density lipoprotein; NAFLD: non-alcoholic fatty liver disease). *p* < 0.05: statistically significant; significant value is shown in bold.

**Table 6:** Correlations of TG/HDL ratio with demographic and clinical parameters.

Parameter	r (95% CI)	*p*
**Age**	0.139 (0.066–0.210)	**<0.001**
**WBCs**	0.183 (0.114–0.246)	**<0.001**
**Hgb**	0.213 (0.140–0.287)	**<0.001**
**PLTs**	−0.008 (−0.083–0.062)	0.818
**Creatinine**	0.174 (0.102–0.237)	**<0.001**
**AST**	0.146 (0.077–0.212)	**<0.001**
**ALT**	0.229 (0.158–0.296)	**<0.001**
**Uric acid**	0.272 (0.208–0.337)	**<0.001**
**Glucose**	0.244 (0.179–0.309)	**<0.001**
**HbA1c**	0.242 (0.168–0.311)	**<0.001**
**LDH**	0.021 (−0.044–0.090)	0.554
**APRI**	0.109 (0.038–0.178)	**0.002**
**FIB-4**	0.061 (−0.010–0.134)	0.084
**ALT/AST ratio**	0.249 (0.183–0.315)	**<0.001**
**TG/glucose ratio**	0.795 (0.763–0.825)	**<0.001**

WBC: white blood cell; Hgb: hemoglobin; PLT: platelet; AST: aspartate aminotransferase; ALT: alanine aminotransferase; LDH: lactate dehydrogenase; APRI: AST-to-platelet ratio index; FIB-4: fibrosis 4 score; TG: triglyceride. *p* < 0.05: statistically significant; significant values are shown in bold.

## Data Availability

The data presented in this study are available on request from the corresponding author.

## Abbreviations

The following abbreviations are used in this manuscript:

NAFLD	Non-alcoholic fatty liver disease
TG	Triglyceride
HDL	High-density lipoprotein
AST	Aspartate aminotransferase
ALT	Alanine aminotransferase
NASH	Non-alcoholic steatohepatitis
US	Ultrasound
CT	Computed tomography
MRI	Magnetic resonance imaging
APRI	AST-to-platelet ratio index
FIB-4	Fibrosis-4
EASL	European Association for the Study of the Liver
ROC	Receiver operating characteristic
WBC	White blood cell
ALP	Alkaline phosphatase
LDH	Lactate dehydrogenase
CRP	C-reactive protein
PLT	Platelet
